# Mechanisms Underlying the Effects of Chloroquine on Red Blood Cells Metabolism

**DOI:** 10.3390/ijms25126424

**Published:** 2024-06-11

**Authors:** Annamaria Russo, Giuseppe Tancredi Patanè, Stefano Putaggio, Giovanni Enrico Lombardo, Silvana Ficarra, Davide Barreca, Elena Giunta, Ester Tellone, Giuseppina Laganà

**Affiliations:** 1 Istituto Comprensivo Primo, 98057 Milazzo, Italy; annamaria.russo81@scuola.istruzione.it; 2 Department of Chemical, Biological, Pharmaceutical and Environmental Sciences, University of Messina, 98166 Messina, Italy; giuseppe.patane@studenti.unime.it (G.T.P.); stefano.putaggio@studenti.unime.it (S.P.); sficarra@unime.it (S.F.); ester.tellone@unime.it (E.T.); giuseppina.lagana@unime.it (G.L.); 3 Department of Medicine and Surgery, University of Enna “Kore”, 94100 Enna, Italy; giovannienrico.lombardo@unikore.it; 4 Virology and Microbiology AOOR Papardo-Piemonte, 98166 Messina, Italy; elenagiunta@live.it

**Keywords:** chloroquine, red blood cells, antioxidant systems, band 3 protein, sulfate transport, hemoglobin, oxygenation–deoxygenation cycle

## Abstract

Chloroquine (CQ) is a 4-aminoquinoline derivative largely employed in the management of malaria. CQ treatment exploits the drug’s ability to cross the erythrocyte membrane, inhibiting heme polymerase in malarial trophozoites. Accumulation of CQ prevents the conversion of heme to hemozoin, causing its toxic buildup, thus blocking the survival of Plasmodium parasites. Recently, it has been reported that CQ is able to exert antiviral properties, mainly against HIV and SARS-CoV-2. This renewed interest in CQ treatment has led to the development of new studies which aim to explore its side effects and long-term outcome. Our study focuses on the effects of CQ in non-parasitized red blood cells (RBCs), investigating hemoglobin (Hb) functionality, the anion exchanger 1 (AE1) or band 3 protein, caspase 3 and protein tyrosine phosphatase 1B (PTP-1B) activity, intra and extracellular ATP levels, and the oxidative state of RBCs. Interestingly, CQ influences the functionality of both Hb and AE1, the main RBC proteins, affecting the properties of Hb oxygen affinity by shifting the conformational structure of the molecule towards the R state. The influence of CQ on AE1 flux leads to a rate variation of anion exchange, which begins at a concentration of 2.5 μM and reaches its maximum effect at 20 µM. Moreover, a significant decrease in intra and extracellular ATP levels was observed in RBCs pre-treated with 10 µM CQ vs. erythrocytes under normal conditions. This effect is related to the PTP-1B activity which is reduced in RBCs incubated with CQ. Despite these metabolic alterations to RBCs caused by exposure to CQ, no signs of variations in oxidative state or caspase 3 activation were recorded. Our results highlight the antithetical effects of CQ on the functionality and metabolism of RBCs, and encourage the development of new research to better understand the multiple potentiality of the drug.

## 1. Introduction

Chloroquine (CQ), a 4-aminoquinoline derivative, was one of the first drugs used in the management and treatment of malaria and inflammatory diseases. It is part of the class of sulfonamides and consists of an aromatic ring to which a side chain is attached, giving the molecule weak base characteristics and the capacity to accumulate in intracellular spaces and lysosomal compartments favoring its pharmacological effects [1,2]. Nowadays, CQ is a valuable anti-autoimmune agent used in rheumatoid arthritis, lupus erythematosus, and in antiviral therapy, with a broad range of activities including HIV and SARS-CoV-2 [3,4,5]. 

The antimalarial activity of the drug is related to its ability to permeate infected red blood cells (RBCs) by simple diffusion, inhibiting the catabolism of heme by the parasite, and thus determining its death. In detail, CQ forms a complex with Hb-derived ferriprotoporphyrin IX, and these highly toxic complexes accumulate in the lysosome, severely limiting the survival of the parasite [6,7]. In addition, CQ can inhibit glutathione-mediated heme degradation, further blocking parasite detoxification [8]. In recent years, with the emergence of the CQ-resistant *Plasmodium falciparum* strain of malaria, the use of the drug has shifted toward the treatment of other diseases. In fact, CQ is used in therapies for the treatment of autoimmune diseases, as the drug enhances regulatory T cells and suppresses effector B cell functions, reducing the severity of the disorder [9,10].

A rich literature on the mild and transient side effects of CQ suggests that the drug is safe, and given its broad spectrum of action, its use is becoming very common even in the absence of malaria disease. In 2020, CQ’s antiviral properties were used against SARS-CoV-2 for the treatment of COVID-19 in many clinical trials in China [4,11,12,13,14]. The antiviral effects of the drug can be attributed to CQ’s ability to hinder viral replication by increasing the endosomal pH [15]. In addition, aminoquinolines, including CQ, hinder the entry of the virus into the cell by interfering with the glycosylation of the angiotensin-converting enzyme 2 (ACE2) and of the viral spike S protein [16]. However, the relatively weak antiviral activity against SARS-CoV-2 and the need for a rapid acquisition of therapeutic dosages in the acute phase of the infection drove the trial to use higher doses than those normally administered for malaria [17,18]. This has caused growing concern about the potential toxicity of the drug, which—although generally safe—may be lethal in overdose, and the margin between the therapeutic and toxic dose is narrow. Specifically, CQ toxicity has been associated with potentially life-threatening cardiovascular disorders, hypotension, arrhythmias, coma, acute respiratory distress syndrome (ARDS), and fatal cardiac arrest [19]. To contribute to expanding the knowledge acquired to date on the effects of CQ, a targeted study on RBCs non-parasitized by *Plasmodium falciparum* was conducted. This study aims to investigate the effect of the drug on healthy RBCs to explore its potential impact on their metabolic behavior. In fact, CQ has a long residence time in the blood, and easily enters RBCs by simple diffusion, where the drug tends to accumulate and bind with cell components [20,21,22]. The analysis was performed by evaluating the influence of high CQ concentrations (10–20 μM) on the metabolic parameters of the RBCs, with a particular focus on Hb and AE1 functionality, caspase 3 and protein tyrosine phosphatase 1B (PTP-1B) activity, the oxidative state of the RBCs, and ATP levels [19]. From the chemical point of view, CQ is a weak base that preferentially concentrates inside the cells, so whole blood instead of plasma or serum is the optimal matrix for pharmacokinetic studies [23]. Therefore, RBCs represent an unparalleled study model because of their direct interaction with the immune cells and their property as an early sensor of several diseases. They must not be considered as simple bags containing hemoglobin and releasing oxygen, and are involved in several metabolic, buffering, and transporter roles. Furthermore, in addition to being the most represented cells in the blood, RBCs are also the simplest, from a metabolic point of view. In fact, the lack of nucleus in mature RBCs limits the network of their metabolic pathways, making them incapable of generating energy via the Krebs cycle. Thus, for energy production and redox homeostasis, these simple cells rely upon an alternating flow of glucose-6-phosphate (G6P) towards the pentose phosphate pathway (PPP) in the high oxygenation state (HOS) or to the Embden–Meyerhof–Parnas glycolytic pathway (EMP) in the low oxygenation state (LOS) [24,25]. This shift of G6P metabolic flux is centered on the T–R states of Hb, where the deoxygenated protein (deoxy-Hb) competitively binds with several glycolytic enzymes (GEs) to the cytosolic domain of the band 3 protein (CDB3). The binding of GEs such as phosphofructokinase, aldolase, and glyceraldehyde-3-phosphate dehydrogenase to CDB3 inhibits—or at least greatly reduces—their catalytic activity. Therefore, in HOS when glycolysis is slowed, G6P may be metabolized by the PPP, ensuring adequate levels of NADPH necessary to protect RBCs from reactive oxygen species (ROS) [26,27]. On the other hand, in LOS when deoxy-Hb binds CDB3, the consequent displacement of GEs results in an increase in the glycolysis pathway, and consequently, an increase in ATP production. The binding of GEs and deoxy-Hb to the CDB3 is also modulated by the phosphorylation state of the AE1 protein. In human RBCs, the regulation of AE1 function involves a balance between the competing activities of protein tyrosine kinases and protein tyrosine phosphatases identified as, respectively, p72(syk) and p56/53(lyn), and PTP1B and SHPTP-2 [28,29,30]. To go into detail, increasing the phosphorylation of the tyrosine residues at positions 8 and 21 in the CDB3 increases the binding of deoxy-Hb to AE1, otherwise provoking GE detachment and activation which results in an increased glycolytic flux at the expense of PPP [24,30]. The caspase 3 protein is part of this metabolic framework; it belongs to the cysteine-aspartate proteases family, responsible for the triggering of the apoptosis cell suicide program. In nucleated cells its autocatalytic activation stimulated by cytosol acidification leads to a protease cascade involving the mitochondrial release of cytochrome c. However, in the enucleated RBCs, caspase 3 activation directly causes the catalytic cleavage of CDB3 and the degradation of cellular proteins. Thus, in RBCs, caspase 3 activity promotes a dramatic metabolic derangement ending up in cell death [31,32,33]. The above provides new avenues for pharmacokinetics investigations that may help to improve the power of CQ.

## 2. Results

To perform a detailed scan on the effects of CQ on healthy RBCs, the drug was tested by evaluating its impact on the two main erythrocyte proteins: Hb and band 3, cytosolic and membrane integral, respectively. 

### 2.1. Influence of CQ on Anion Exchange 

The influence of CQ on AE1, studied by spectrophotometric evaluation of anion exchange kinetic values, shows a concentration-dependent trend of anion transport activity in the investigated range (from 2.5 to 20 μM). CQ affects AE1 by increasing the rate of anion exchange across the membrane, starting already at a concentration of 2.5 μM, reaching its maximum effect at the higher tested concentration of 20 μM. The rate constant varies in the absence and in the presence of CQ, from 0.012 to 0.027 min^−1^, respectively. 

**Figure 1:**
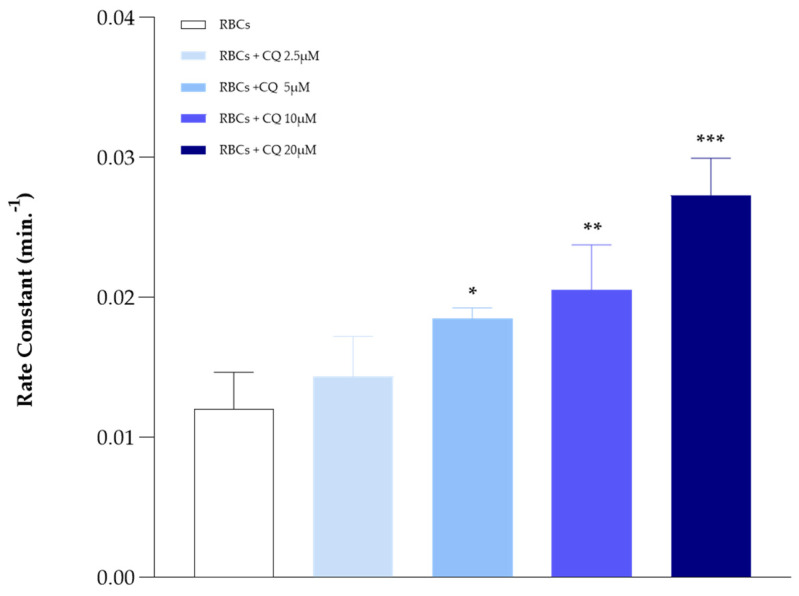
Sulfate flux measured in oxygenated RBCs in the absence and presence of increasing CQ concentrations (2.5, 5, 10, and 20 μM). Values are presented as the mean ± SD (N = 5), * *p* < 0.05 vs. RBCs; ** *p* < 0.01 vs. RBCs; *** *p* < 0.0001 vs. RBCs.

**Figure 2:**
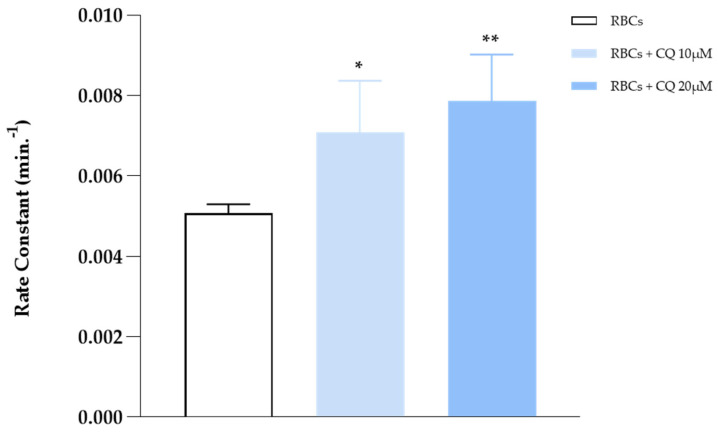
Sulfate flow measured in deoxygenated RBCs in the absence and presence of CQ (10 and 20 μM). Values are presented as the mean ± SD (N = 5), * *p* < 0.05 vs. RBCs; ** *p* < 0.01 vs. RBCs.

As shown in Figure 1, AE1 activity tends to be amplified as the drug amount increases, up to a maximum of 20 μM. Beyond this threshold value, the drug loses its effect. To assess whether this condition was respected even in LOS, RBCs were deoxygenated and then incubated with CQ at concentrations of 10 and 20 μM. The results shown in Figure 2 document that, although less evident, the drug also stimulates anion exchange in this condition (∼15% saturation), shifting the rate constant values from 0.0052 to 0.0075 min^−1^ in the absence and presence of CQ (10 and 20 μM), respectively. The experiments showed an anion flow increase of 50% in HOS and 30% in LOS, respectively.

### 2.2. CQ Influence on Hb Structure and Function 

**Figure 3:**
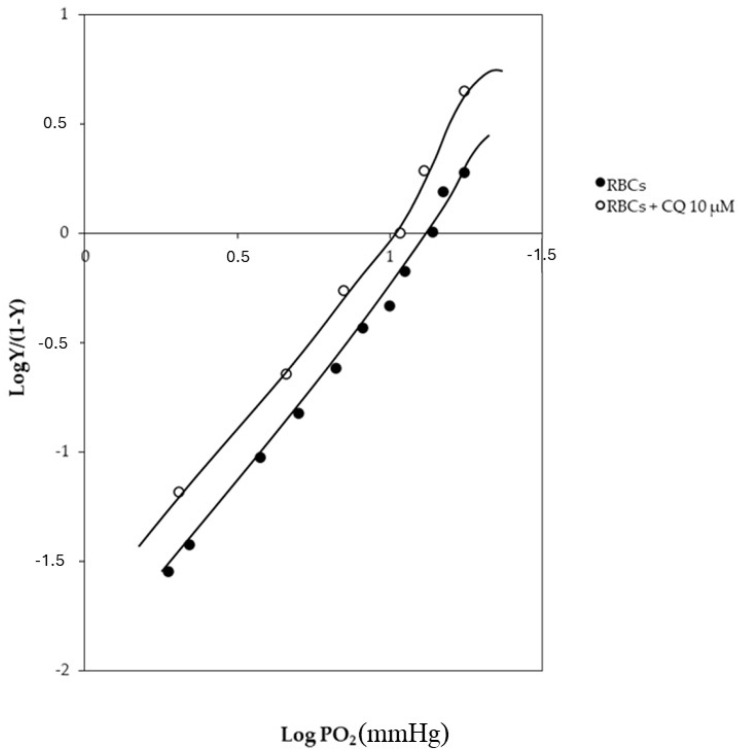
Hill plot for the binding of oxygen to Hb in the absence (closed symbols) and in presence (open symbols) of 10 μM CQ. Conditions: 0.1 M HEPES buffer plus 0.1 M NaCl and 3 mM 2,3-biphosphoglycerate at pH 7.4 and 37 °C.

Recalling the well-known functional interaction between AE1 and Hb, a set of experiments were conducted to test how CQ effects the oxygen affinity of Hb [34]. As shown in Figure 3, the drug slightly increases the oxygen affinity properties of Hb by shifting the curve on the left (log P_50_ = 1.209 and 1.033 in the absence and presence of CQ, respectively). 

**Figure 4:**
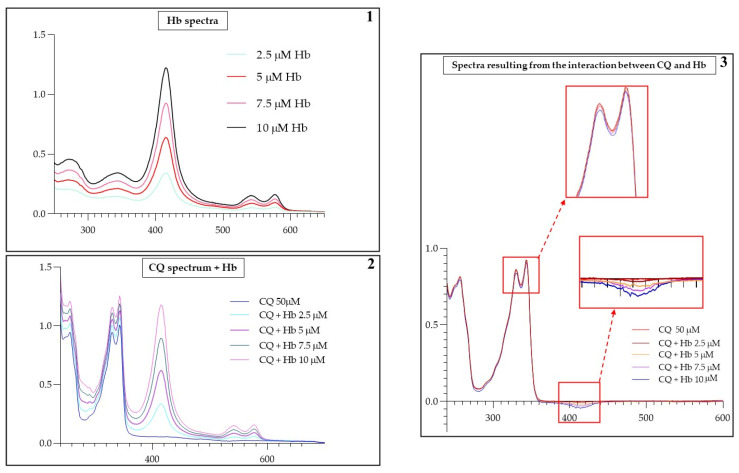
UV-visible absorption spectra of CQ (50 µM) at pH 7.4, in the absence or presence of increasing Hb concentrations (2.5 µM, 5 µM, 7.5 µM, 10 µM). The image shows an illustration of the variation of absorbance obtained with several different experiments. Section 1: shows the spectrum of Hb at different concentrations (2.5, 5, 7.5, 10 μM). Section 2: shows the CQ (50 μM) spectrum variation by adding increasing concentrations of Hb. Section 3: shows the CQ (50 μM) spectrum minus Hb spectra (5, 7.5, 10 μM).

Potential CQ–Hb interaction was also evaluated by spectrophotometric analysis. The results shown in Figure 4 display the typical absorption peak of CQ at 343 nm, corresponding to the aminoquinoline ring. Following the addition of increasing concentrations of Hb (0–10 µM), there are no significant changes in the band shapes but it is evident a light decrease in the maximum of absorbance region at 343 nm and 405 nm, the last corresponding to the Soret band characteristic of heme groups. This hypochromism suggests the existence of a weak CQ-Hb interaction involving the heme aromatic ring and the aminoquinoline ring of the drug.

### 2.3. CQ Influence on RBCs Oxidative State

**Figure 5:**
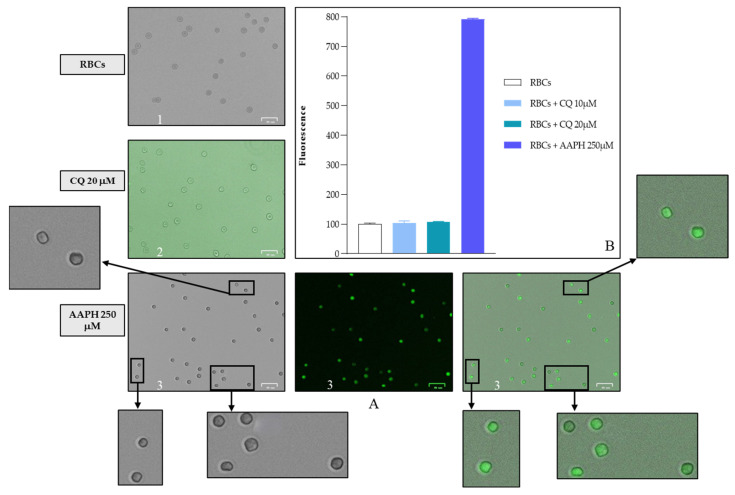
Analysis of the effect of 0 and 20 µM CQ and 250 µM AAPH on ROS generation/oxidative stress calculated from fluorescence emission (section **B**). Representative morphological images of the changes induced by AAPH (3), or CQ (2) and no-oxidant treatments (1) (section **A**). Values are presented as the mean ± SD (N = 5). Scale bar: 25 µm.

The influence of CQ on Hb oxidation state and RBC membrane integrity was further analyzed by incubating fresh RBCs for two hours with different concentrations of CQ (2.5, 5, 10, 20, and 30 μM) at 37 °C. It can be concluded that the drug does not affect the Hb redox state because no increased MetHb values were observed. Similarly, there were no alterations in hemolysis percentage, which was within 3% in all tested CQ concentrations. The redox state of RBCs was also tested by quantifying ROS generation using a DCFH-DA fluorescent probe (see Figure 5). Interestingly, there was no increase in fluorescence between the control condition and the addition of CQ to the wells, unlike in the positive control with the free radical-generating azo initiator 2,2′-azobis (2-methyl-propanimidamide) dihydrochloride (AAPH).

**Figure 6:**
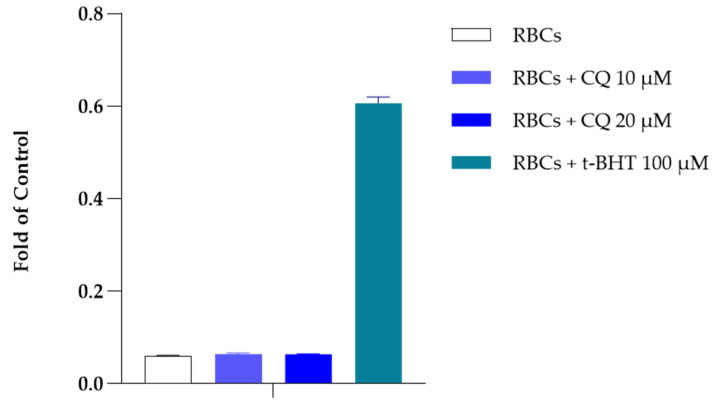
Caspase 3 activity in RBCs in the absence and in the presence of CQ (10 and 20 µM). Results are from four independent experiments ± standard deviation.

Morphological analysis under light microscopy of RBCs incubated for 60 and 90 min with 20 μM CQ did not show significant morphological alterations. After 24 h of incubation, erythrocytes treated with CQ showed negligible altered morphology, with few irregular shapes. These results were confirmed by caspase 3 inactivation determined in the presence of CQ and in the presence of 100 μM of tert-butyl-hydroperoxide (t-BHT), a well-known oxidant agent [35]. As shown in Figure 6, the data observed from incubating the RBCs in the absence and presence of CQ (10 and 20 µM, respectively) indicate the inability of CQ to mediate the stimulation of caspase 3.

Since caspase 3 activity has a critical role in inducing RBC apoptosis through the catalytic cleavage of CDB3, we may conclude that the presence of CQ at the various tested concentrations and incubating times does not induce AE1 proteolysis. 

Besides, the scenario deriving from DCFHA, MetHb, and hemolysis experiments indicate a substantially stability of the redox state of the RBCs that if altered towards the oxidation should drive to the activation of caspase 3 [32,36,37].

### 2.4. CQ Influence on ATP Levels and PTP-1B Activity 

**Figure 7:**
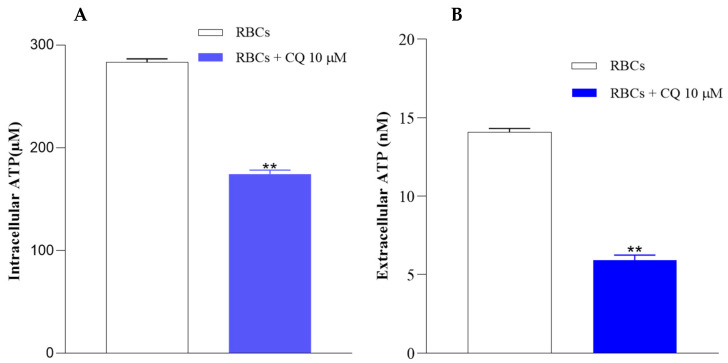
Effect of CQ on the intracellular (**A**) and extracellular (**B**) ATP levels in RBCs. ATP concentrations were measured at the end of the incubation time of RBCs without and with 10 μM CQ. Results are from four independent experiments ± standard deviation. Asterisks indicate significant differences at *p* < 0.05 versus control. Values are presented as the mean ± SD (N = 5). ** *p* < 0.05 compared with control.

It is known that Hb conformational states are correlated with the release of ATP from the RBCs. Therefore, the influence of CQ on intracellular ATP levels and ATP release from the cells was evaluated [38,39]. The results shown in Figure 7 highlight significantly lower levels of intracellular ATP in RBCs pretreated with 10 µM CQ compared to erythrocytes under normal conditions (A); the same trend is visible in panel B where a decrease in the amount of ATP released from RBCs in the presence of CQ with respect to the control is shown. 

Specifically, a decrease of 62.7% and 47.6% of intra and extracellular ATP levels was registered, respectively, with respect to the control (RBCs in absence of CQ).

**Figure 8:**
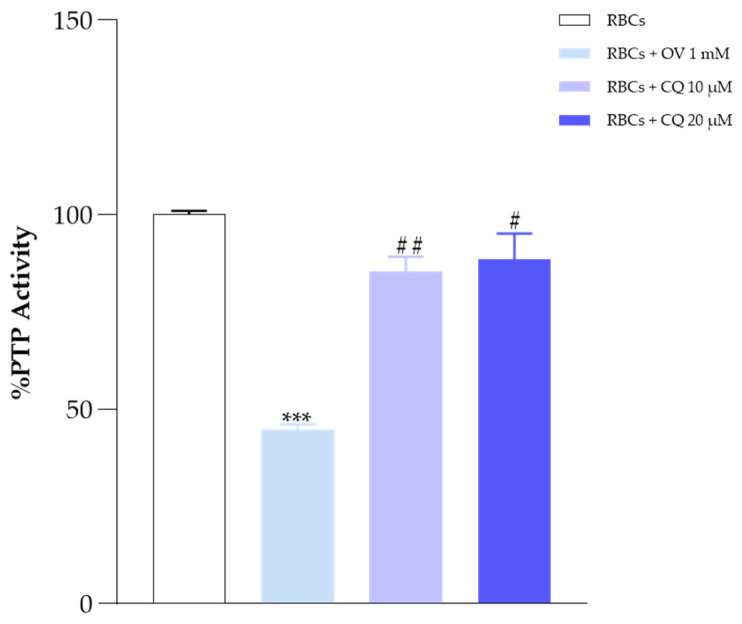
Phosphatase activity in normal human RBCs, incubated in the absence or in the presence of 10–20 μM of CQ. Values are the mean ± SD of at least three different experiments. *** *p* < 0.005, ## *p* < 0.05 and # *p* < 0.5 compared with control.

ATP is an activator of PTP-1B, the main RBC membrane-associated phosphatase. PTP-1B activity could be affected by reduced ATP levels; to test this, a set of experiments were carried out using orthovanadate (OV), a well-known phosphatase inhibitor. Figure 8 clearly shows a slight decrease in PTP-1B activity in RBCs incubated both with 10 and 20 μM CQ compared with OV (1 mM) [40,41]. 

In RBCs, the process of phosphorylation/dephosphorylation of tyrosine residues of membrane proteins is implicated in the regulation of several functions, including the regulation of metabolism, transport across membranes, redox stress, cell shape, and death [42,43]. AE1 is finely regulated in vivo by the concerted action of two proteins: the tyrosine kinase Src and the protein phosphatase PTP-1B, associated both structurally and functionally with the AE1 protein. CQ’s slight inhibition of phosphatase activity and the reduced ATP levels might contribute to destabilizing the RBC’s phosphorylation state and trigger an unfavorable metabolic condition for the cell. However, it is interesting to note that the experimental data do not show harmful oxidative indices (no hemolysis, no meta-Hb, no intracellular ROS, and no morphological changes).

### 2.5. CQ Effect on the Erythrocyte Membrane

**Figure 9:**
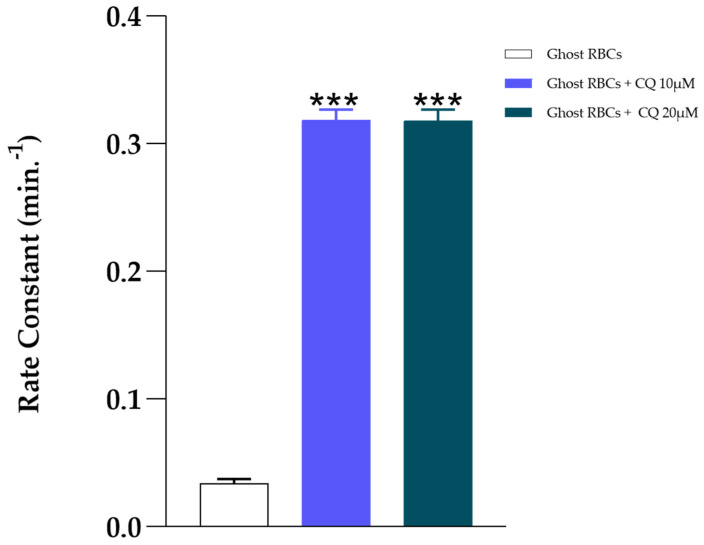
Effect of CQ (10 and 20 μM) on ghosts incubated at 37 °C and pH 7.4. Values are the mean ± SD of at least three different experiments. *** *p* < 0.005 compared to the control.

To evaluate the direct effect of the drug on the erythrocyte membrane, a series of kinetic experiments on anion transport were performed on ghosts, i.e., RBCs completely devoid of their intraerythrocytic content. Ghosts were incubated for 60 min at 37 °C and pH 7.4 in the absence and presence of CQ. After the incubation time, the anion flux was measured; the results are shown in Figure 9, and confirm the stimulating effect of the drug on anion transport, with a rate constant value of 0.034 min^−1^ and 0.24 min^−1^ in the absence and presence of CQ, respectively.

## 3. Discussion

CQ crosses the erythrocyte membrane by passive diffusion, and in accordance with Jagger et al., our results show a direct correlation between Hb oxygenation states and the ATP release from RBCs. The drug’s gradual interaction with Hb contributes to the alteration of some metabolic parameters, including exchanger activity and erythrocyte metabolic fluxes [21,22,38]. In RBCs, the reduced intracellular levels of ATP induced by the presence of CQ can be correlated with an alteration of the phosphorylation balance, a hypothesis strongly supported by our experimental data highlighting the inhibition of PTP-1B. In this regard, it is useful to remember that, in line with our results, low intracellular ATP levels can result in the inhibition of PTP-1B activity and that cells subjected to energy depletion show detachment of PTP-1B from AE1 [40]. Thus, the experimental results lead us to postulate the existence of a direct action of the drug on the membrane of RBCs.

**Figure 10:**
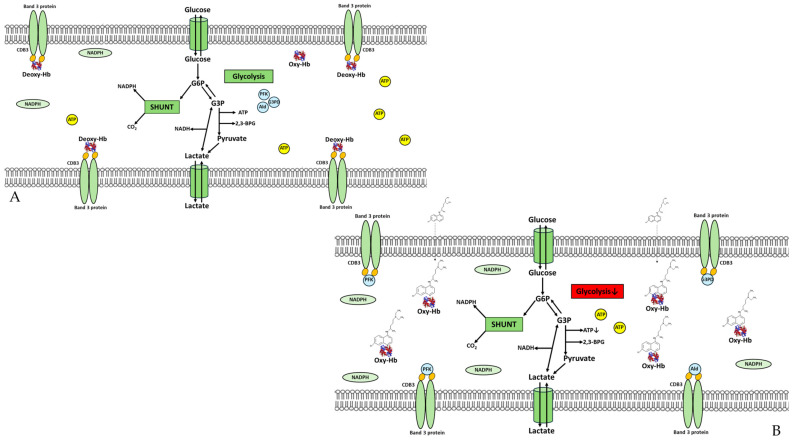
Schematical representation of RBC metabolism in the absence (**A**) and presence (**B**) of CQ.

Moreover, the reduced ATP intracellular availability may itself be the cause of the decreased ATP release from the RBCs. In fact, ATP released from RBCs is closely linked to its production (through the glycolysis pathway in RBCs) in turn, dependent on the oxygenation states of Hb. In RBCs, the modulation of G6P metabolic fluxes towards glycolysis or PPP is regulated by the oxygenation states of Hb through its binding with CDB3. From a metabolic point of view, the presence of CQ could favor the binding of GEs to CDB3, causing their inhibition and the consequent slowing down of the glycolytic pathway with a decrease in ATP synthesis (see Figure 10). The reduction in the glycolysis pathway due to the presence of CQ is also supported by the drug’s weak inhibition of lactate dehydrogenase [44]. Furthermore, the CQ–Hb interaction stabilizing the R conformational state of the molecule and alleviating the competition between GEs and deoxy-Hb for CDB3 causes a decrease in the structural hindrance of the AE1 channel, increasing the activity of the anion exchanger [45]. In line with the above, our experimental data show an increase in the anion flux rate in RBCs incubated in the presence of the drug, and the evidence from our experiment with ghosts suggests a direct interaction of CQ with the phospholipid bilayer. After all, CQ is a cationic amphiphilic drug, and as such, binds to biological membranes. The binding, stabilized by nonspecific hydrophobic and ionic interactions, can potentially affect many functions of the membrane by altering its structure and functions [46,47,48,49]. In addition, CQ being a weak base decreases the hydrogen ionic concentration, favoring cytosolic alkalinization, which in turn contributes to increasing the anion flux. To elaborate, regarding the structure of AE1, a decrease in pH generates an H bridge between the residues 752 and 699, resulting in a decrease in anion transport; the presence of CQ causes the disruption of this H bridge and the consequent increasing of anion flux [50]. Overall, our data seem to indicate a change in the utilization of metabolic fluxes, which can be interpreted in a more complex and global physiological and regulatory mechanism of CQ’s adaptation of RBCs.

In a broader view, the functional role of CQ can be seen as an antagonist of physiological vasodilation. By reducing the amount of ATP released by the RBC, CQ reduces the paracrine action of the nucleotide on the purinergic receptors present on the vascular endothelium. In detail, RBCs moving passively in the blood circulation actively interact with the blood vessel in response to the oxygen tension of the tissues promoting vasodilatation. These interactions occur between the ATP released by the RBCs and the purinergic receptors present on the vascular endothelium, causing the synthesis and release of nitric oxide (NO). The diffusion of NO into adjacent smooth muscle cells results in vasodilation. In the presence of CQ, the reduced ATP synthesis and release from RBCs leads to a decrease in NO synthesis and diffusion, which in turn negatively affects vasodilation input. This hypothesis is partly confirmed by Perecko et al. who showed that both chloroquine and its hydroxyderivative, hydroxychloroquine, inhibited NO production in the immortalized mouse macrophage cell line RAW 264.7 and mouse bone marrow-derived macrophages [51,52]. The above reports open new points of research on the side effects of the drug, especially in patients with coronary or cardiovascular diseases. In these pathological conditions the alteration of vasodilation due to CQ treatment could lead to a worsening of the disease, especially for patients subject to tachycardia or bradycardia phenomena which could be significantly affected by a high dosage of the drug. In cardiovascular diseases, impaired NO synthesis, caused by a high dosage of CQ, may increase the risk factor for major adverse cardiovascular events. This is due to the inhibitory action of NO on platelet and leukocyte aggregation, infiltration of inflammatory cells, and proliferation of vascular smooth muscle cells [53,54]. Moreover, endogenous NO levels are closely related to those of angiotensin II, a potent vasoconstrictor. The balance between these two modulators plays a key role in the pathogenesis of the cardiovascular system [55,56]. Inhibition or suppression of one increases the activity of the other, so the inhibitory effect of CQ on NO synthesis could cause an exacerbation of vasoconstriction produced by angiotensin II [57,58]. Treatment with high CQ dosage could also cause damage in patients with essential hypertension. In these cases, the reduced NO bioavailability could compromise endothelial function, worsening the already existing state of hypertension. In this regard, several studies demonstrating the link between an impaired nitric oxide production pathway and the onset of essential hypertension may be useful [59,60,61]. 

The results discussed so far show an unfavorable metabolic pattern; however, it is interesting to note that in apparent contrast, the experimental data do not show the presence of any evident oxidative stress. On the one hand, this condition may be explained by the increase in anionic flux favoring the rapid removal of CO_2_ and peroxy-nitrite, contributing to the prevention of secondary radical generation (such as carbonates and nitrogen dioxides) [62,63,64]. On the other hand, the shift of the Hb conformational state towards the R state caused by CQ leads to a reduced affinity between oxy-Hb and CDB3 that finally causes an increase in bound GE-CDB3. Therefore, this condition reducing the G6P flux towards the glycolysis pathway makes it available to be metabolized in the PPP, ensuring adequate levels of NADPH necessary to protect RBCs from ROSs which may eventually be present [26,27]. Moreover, as mentioned previously, since CQ is a weak base, it tends to promote alkalinization of the cytosol, which promotes the maintenance of caspase 3 in the form of inactive zymogen procaspase 3 [35]. 

In conclusion, our results, which highlight the antithetical effects of CQ on RBC functionality and metabolism, could also be interpreted as a larger metabolic response with several environment-related input stimuli. What emerged from the study encourages new research to better understand the drug’s multiple potentiality.

## 4. Materials and Methods

### 4.1. Reagents and Compounds

All reagents were purchased from Sigma Aldrich (St. Louis, MO, USA). Human blood was collected from informed healthy volunteers aged 27–30 years. RBCs were collected in tubes with EDTA or heparin (used as anticoagulants) and used fresh for the experiments. The study was approved by a Local Ethics Committee (prot. 71-23 del 5 April 2023) in accordance with the Helsinki Declaration. A stock solution of chloroquine was prepared using NaCl 0.9% as buffer.

### 4.2. Preparation of Erythrocytes

Collected fresh blood samples, with heparin-like anticoagulants were washed three times with an isosmotic 0.9% NaCl solution and treated as previously reported [65]. During washing, the samples were centrifuged at 1500 rpm for 10 min at 4 °C and the supernatant was discarded from the pellet. After washing, the RBCs were resuspended in the 20 mOsmol 2-[4-(2-hydroxyethyl) piperazin-1-yl]ethanesulfonic acid (HEPES) incubation buffer and it was adjusted to pH 7.4, as measured by an Omostat OM-6020 apparatus (Daiichikagakuco, Kyoto, Japan). All the experiments were conducted with a hematocrit percentage of 3%, except for the fluorescence assay, which was done at a percentage of 1%. See Table 1 for a complete overview of incubation times and hematocrit percentage.

In the experiments with deoxygenated RBCs, a buffer with a pH 0.1 lower than previously used was utilized to compensate for the Haldane effect that takes place during deoxygenation of RBCs, as reported in the literature [66]. The degree of deoxygenation achieved ranges from 15 to 90%, and Hb saturation during the experiments was recorded by spectrophotometric reading in the spectral range (450–700 nm), using a Beckman DU 640 (Harbor Boulevard, Fullerton, CA, USA), as reported by Zijlstra et al. [67]. The degree of hemolysis and the levels of methemoglobin formed, during incubation with different concentrations of chloroquine, were recorded at the end of the incubation time, as reported in the literature by Tellone and collaborators [68].

#### Methemoglobin (MetHb) Determination

After washing, red blood cells were incubated with CQ (10 and 20 μM), for 24 h, lysed with cold distilled water, and frozen. After the incubation time they were centrifuged at 18,000 rpm for 30 min. The percentage of MetHb was determined by spectrophotometric analysis, taking the wavelength range of 500–680 nm. The MetHb content never exceeded 2% under any of the experimental conditions, so it is considered not significant. 

### 4.3. Effect of Chloroquine on Erythrocyte Hemolysis

After incubating samples with various concentrations of chloroquine (from 2.5 to 20 μM), we assessed its safety by determining the percentage of hemolysis through spectrophotometric analysis. In detail, the measurement of the degree of hemolysis was done spectrophotometrically at 576 nm by taking the ratio of Hb released from the cells to the total Hb contained in the cells after total hemolysis with ultrapure water. At the end of the readings, hemolysis levels were calculated using the following formula:

H (%) = A/B × 100%

where H (%) represents the percentage of hemolysis achieved, A is the level of hemoglobin released from samples incubated with or without different concentrations of chloroquine, and B represents the maximum hemoglobin that is released following total hemolysis with ultrapure water. There are no alterations in hemolysis percentage which was within 3% in all sample tested.

### 4.4. Morphological Analysis of the Treated Red Blood Samples

After different incubation times, packed erythrocytes samples were collected and washed three times with HEPES washing buffer to remove unbound chloroquine during treatment. During each step, the samples were centrifuged at 2500 rpm for 3 min at 4 °C and the supernatant was removed. Then, the images of treated collected red blood cells were captured using a Zoe Fluorescent Cell Imager (Bio-Rad Laboratories, USA). Following dilution with HEPES buffer, a small amount of the treated blood samples was carefully applied onto freshly cleaved mica to form a single layer and utilized for morphological analysis.

### 4.5. Measurement of Oxygen Dissociation Curves (ODC)

To obtain the oxygen–hemoglobin affinity data, purified hemoglobin must be obtained. Briefly, to purify hemoglobin, the samples were lysed in ice distilled water and diluted with 0.1 M NaCl and 0.1 M HEPES buffer at pH 7.4. The obtained hemolysate was passed through a Sephadex G-25 column. Samples with and without chloroquine treatment were analyzed at different concentrations. In particular, the binding affinity between oxygen and purified hemoglobin was investigated using samples treated with 10 μM chloroquine. As reported in the literature, we obtained the partial pressure of the ligand at which 50% of the heme is oxygenated (P_50_) and the Hill coefficient (n), an empirical constant, at 37 °C, using 2,3-diphosphoglycerate (3 mM) as a physiological allosteric modulator, using the tonometric method [69]. An average standard deviation of 8% was calculated for the P_50_ values. 

### 4.6. Uv-Visible Spetroscopy

To evaluate the interaction between purified hemoglobin and chloroquine, UV-Visible spectroscopy was performed as reported by Patanè et al. [70]. The analysis was performed on a solution of chloroquine with a constant concentration of 50 μM. We added increasing amounts of hemoglobin purified up to give a final concentration of 10 µM while reading the changes in absorbance in the wavelength range of 200–600 nm, with a 1 cm optical path of the quartz cuvette. Then, the spectra of the pH 7.4 HEPES buffer, alone or combined with the equivalent concentration of purified hemoglobin used in the titration, were subtracted from those of the respective sample solutions under identical experimental conditions. This experiment was repeated three times, and the data are presented as the mean ± SD.

### 4.7. Fluorescence Assay

To verify that the different tested concentrations of chloroquine did not cause oxidative stress, we evaluated ROS production by DCFH-DA fluorometric assay. Specifically, in this assay, we take advantage of the fact that DCFH-DA is not polar or ionic; therefore, it is well able to cross the membrane of RBCs and is subsequently deacetylated by cytoplasmic esterases to DCFH, and then in the presence of ROS it is oxidized to fluorescent DCF. Erythrocytes were processed as described in the literature by Bardyn and coworkers, with minor modifications [71]. Briefly, after discarding the serum and white blood cells, fresh erythrocytes were resuspended in 0.9% NaCl until a 10% hematocrit was obtained and incubated for 30 min at 37 °C with DCFH-DA to allow the probe to correctly enter inside the RBCs. At the end of the incubation time, we centrifuged the samples at 3000 rpm for 5 min to remove the unincorporated probe inside the erythrocytes. Thus, 20 μL of our samples were placed in the 96-well imaging plates and diluted to a hematocrit of 1% in a final volume of 200 μL, using NaCl 0.9% as buffer, AAPH 250 μM for positive control, and the different concentrations of chloroquine (10, 20 μM) as treatment. After two hours of incubation time at 37 °C, the detection of ROS was assessed using the fluorometer “FluoroStar omega”, with an excitation length at 492 nm and an emission length at 527 nm.

### 4.8. Kinetic Measurements

The erythrocytes were incubated in the working buffer (35 mM Na_2_SO_4_, 90 mM NaCl, 25 mM HEPES buffer, and 1.5 mM MgCl_2_), adjusted to pH 7.4 at 25 °C with different concentrations of chloroquine, as reported in the literature [72]. At different time intervals (5, 15, 30, 60, 90, and 120 min), 10 µM of 4-acetamido-40-isothiocyanostilbene-2,20-disulfonic acid (SITS) was added to each tube containing the erythrocyte suspension to stop the reaction. Then, the packed cells were separated from the incubation medium buffer by centrifugation at 10 min at 4000 rpm (4 °C) with a J2-HS Centrifuge, Beckman, and washed three times at 4 °C to remove the sulfate left out of the cells. Following the final washing step, the erythrocytes were lysed using perchloric acid (4%). To separate the membranes from the rest of the supernatant, the lysed cells were centrifuged for 10 min at 4 °C at 4000 rpm. Then, we added a mixture of glycerol and distilled water (1:1, v/v), 4 M NaCl, 1 M HCl, and 1.23 M BaCl_2_⋅2H_2_O to eliminate sulfate ions from the supernatant and to achieve a homogeneous barium sulfate precipitate. The absorbance of this suspension was then measured within the range of 350–425 nm. The sulfate concentration was determined using a calibrated standard curve, established by measuring the absorbance of suspensions containing known quantities of sulfate [73]. Experimental data on sulfate concentration over time were analyzed using the following equation:

𝑐(𝑡) = 𝑐∞(1 − 𝑒−𝑘𝑡)

where *c*(*t*) is the sulfate concentration at time *t*, *c*∞ is intracellular sulfate concentration at equilibrium, and *k* is the rate constant of sulfate influx.

### 4.9. Caspase 3 Assay

To assess caspase activity, fresh blood samples were washed three times with an isosmotic 0.9% NaCl solution and processed as reported in the literature by Tellone and collaborators [74]. Briefly, after discarding the serum and white blood cells, the erythrocytes were resuspended in a maintenance buffer (35 mM Na_2_SO_4_, 90 mM NaCl, 25 mM HEPES, and 1.5 mM MgCl_2_) at pH 7.4, to obtain a 3% hematocrit. Then, they were incubated for two hours, without and with tert-butyl-hydroperoxide (t-BHT) 100 μM and with different concentrations of chloroquine. At the end of the incubation time, the samples were centrifuged and resuspended in HEPES buffer. Then, they were lysed by sonication, and to obtain partial purification of caspase 3, the lysates were centrifuged at 15,000 rpm for 10 min and the supernatant was filtered through a Microcon YM 30 (nominal molecular weight limit 30,000). The final product of each sample was incubated for 1h at 37 °C with the caspase 3-specific substrate AcDEVD-pNA and adjusted to volume with 100 mM HEPES buffer. The release of pNA, the product of this colorimetric reaction, was analyzed by spectrophotometry at 405 nm and correlated with caspase 3 activity.

### 4.10. ATP Measurement9

#### 4.10.1. Measurement of Intracellular ATP

Intracellular levels of ATP were measured by the luciferin–luciferase method, as reported by Tellone et al. The amount of light emitted is directly proportional to the ATP detected inside the samples. Briefly, treated erythrocytes without and with CQ (10 μM) were diluted and incubated for 1 h with Mastoparan 7 (Mas 7), one of the most common inducers of the Gi protein. Then, to block further ATP formation, the samples were deproteinized and diluted with trichloroacetic acid (TCA). Finally, after centrifuging at 3000 rpm at 4 °C for 10 min, a solution of D-luciferin and Firefly Lantern Extract (FLE 250) was added to the samples at a ratio of 1:1. The emitted light was recorded using a Bio Orbit 1251 luminometer (Bio-Orbit Oy, Turku, Finland) [75].

#### 4.10.2. Measurement of Extracellular ATP

Extracellular ATP levels were determined by the luciferin–luciferase method, as reported by Tellone et al. After treating the red blood cells, as reported in the previous subparagraph, we collected the supernatant (10 μL) and diluted it with distilled water (990 μL). We picked up 100 μL to which we added D-luciferin and Firefly Lantern Extract (FLE 250) in a ratio of 1:1. The emitted light was recorded using a Bio Orbit 1251 luminometer [75].

### 4.11. Determination of Phosphatase PTP-1B Activity

One of the major systems of regulation of AE1 happens through the kinase and phosphorylase system. Among the phosphatases that are most present in erythrocytes is PTP-1B, which is structurally related to band 3. To understand the influence of CQ on the phosphorylation status of AE1, we performed the following assay as described by Maccaglia et al., with minor modifications. Specifically, samples treated without and with CQ (10, 20 μM), were compared with data obtained from samples incubated with OV (1mM), a known phosphatase inhibitor and using p-nitrophenyl phosphate (p-NPP) as substrate. Briefly, the membranes of the treated samples were resuspended in 25 mM HEPES buffer containing 0.1 mM phenylmethanesulfonylfluoride (PMSF), 20 mM MgCl_2_, and 15 mM p-NPP, and incubated at 37 °C for 60 min. After centrifugation, the release of p-nitrophenol was detected at 410 nm [75,76].

### 4.12. Preparation of RBCs Ghosts

After washing, red blood cells were lysed with ice-cold hypotonic buffer consisting of 5 mM Tris and 5 mM KCl. The lysed red blood cells were centrifuged at 15,000 rpm for 10 min at 4 °C; then the supernatant, containing the intracellular components, was eliminated. Subsequently, after incubation at 37 °C for one hour, the membranes were resealed, and the solubilized plasma membranes were conserved at −80 °C.

## Figures and Tables

**Table 1:** Experimental conditions.

Experiment	Incubation Time	Hematocrit %
Hemolysis %	24 h	3%
Methemoglobin	24 h	3%
Morphological analysis	1 h; 1:30 h; 24 h	1%
Fluorescence assay	2 h	1%
Kinetic measurements	90 min	3%
Caspase 3 assay	2 h	3%
Intra/extracellular ATP	1 h	3%
Phosphatase PTP-1B activity	1 h	3%
RBC ghosts	1 h	3%

## Data Availability

The data that support the findings of this study are available from the corresponding author upon reasonable request.

## Abbreviations

Chloroquine (CQ); human immunodeficiency virus (HIV); red blood cells (RBCs); severe acute respiratory syndrome coronavirus 2 (SARS-COV-2); angiotensin-converting enzyme 2 (ACE2); acute respiratory distress syndrome (ARDS); adenosine triphosphate (ATP); hemoglobin (Hb); Nicotinamide adenine dinucleotide phosphate (NADP); glucose-6-phosphate (G6P); pentose phosphate pathway (PPP); high-oxygenation state (HOS); Embden–Meyerhof–Parnas (EMP); low-oxygenation state (LOS); protein tyrosine phosphatase 1B (PTP-1B); deoxygenated hemoglobin (deoxy-Hb); glycolytic enzymes (GE); cytoplasmatic domain of the band 3 protein (CDB3); N-(2-hydroxyethyl)-piperazine-N1-2-ethanesulfonic acid (HEPES); methemoglobin (MetHb); P_50_ (partial pressure of the ligand at which 50% of hemes is oxygenated); n (Hill coefficient); reactive oxygen species (ROS); 2,2′-azobis(2-amidinopropane) dihydrochloride (AAPH); p-nitrophenyl phosphate (p-NPP); phenylmethanesulfonylfluoride (PMSF); standard deviation (SD); band 3 (B3); orthovanadate (OV); cytoplasmatic domain of band 3 (CDB3); nitric oxide (NO); Mastoparan 7 (Mas 7); trichloroacetic acid (TCA).
